# Natural Products as Tools for Defining How Cellular Metabolism Influences Cellular Immune and Inflammatory Function during Chronic Infection

**DOI:** 10.3390/v7122933

**Published:** 2015-11-30

**Authors:** Erica S. Lovelace, Stephen J. Polyak

**Affiliations:** 1 Department of Laboratory Medicine, University of Washington, Seattle, WA 98195, USA; elove@uw.edu; 2 Department of Microbiology, University of Washington, Seattle, WA 98195, USA; 3 Department of Global Health, University of Washington, Seattle, WA 98195, USA

**Keywords:** natural product, inflammation, HCV, HIV, mTOR, NF-κB, AMPK

## Abstract

Chronic viral infections like those caused by hepatitis C virus (HCV) and human immunodeficiency virus (HIV) cause disease that establishes an ongoing state of chronic inflammation. While there have been tremendous improvements towards curing HCV with directly acting antiviral agents (DAA) and keeping HIV viral loads below detection with antiretroviral therapy (ART), there is still a need to control inflammation in these diseases. Recent studies indicate that many natural products like curcumin, resveratrol and silymarin alter cellular metabolism and signal transduction pathways via enzymes such as adenosine monophosphate kinase (AMPK) and mechanistic target of rapamycin (mTOR), and these pathways directly influence cellular inflammatory status (such as NF-κB) and immune function. Natural products represent a vast toolkit to dissect and define how cellular metabolism controls cellular immune and inflammatory function.

## 1. Introduction

Natural products are pure compounds or mixtures, and they are often derived from primary and secondary metabolites of cells, tissues, and secretions from microorganisms (bacteria, archae, fungi), plants, and animals. They are found in marine and terrestrial environments. Pharmacognosy is the study of natural products, which is studied throughout the world through organizations such as the American Society of Pharmacognosy [1] and the Gesellschaft für Arzniepflanzen (GA society) [2].

Two-thirds of the population of the world uses alternative therapies, including botanical medicines, as their primary source of health care [3]. Natural products continue to play a critical role in drug discovery; a significant portion (28%) of the new drugs approved between 1981 and 2010 were of natural product origin [4]. Moreover, the 2015 Nobel Prize in Medicine was awarded to three scientists who discovered Invermectin and Artemisinin, two natural products with potent anti-infective properties. Thus, natural products represent a vast chemical toolbox of potent medicines with demonstrated health-promoting actions. However, a consistent issue with research on natural products, with the exception of a few drugs, is the lack of clarity surrounding their mechanisms of action. Following ingestion and absorption, natural products produce biological responses in mammalian cells by several mechanisms including direct antioxidant functions, interaction with membranes, and alterations in mitochondrial function, signal transduction, and gene expression. This review will focus on the emerging evidence that polyphenolic natural products alter cellular metabolism, which has profound impacts on cellular inflammatory status and function, and may represent a mechanistic underpinning for why so many polyphenolic natural products display variable but consistent anti-inflammatory actions *in vitro* and *in vivo*.

## 2. Inflammation

Inflammation is a homeostatic response to infection or cellular/organ damage. It involves an initial sensing event, which is amplified by cellular signaling within the damaged tissue. Pro-inflammatory cytokine and chemokine production recruits immune effector cells to remove pathogens and repair the damage. Once the injury/infection is eliminated, inflammation subsides through multiple feedback mechanisms. Collectively, the processes of sensing, amplification, and resolution represent the “good” side of inflammation. However, there is a “bad” side to inflammation, when inflammatory processes persist. In the proceeding section, we describe the process of chronic inflammation and its association to disease in the context of HCV and HIV infections.

## 3. Chronic Inflammation: The Case for HCV

Hepatitis C is a global medical problem that causes significant liver disease in millions of people. The hepatitis C virus (HCV) displays propensity for chronic infection and causes severe liver disease. Despite huge strides in directly acting antiviral (DAA) drugs that rapidly cure HCV infection, there will be a lag in the global eradication of HCV because of the high cost of therapy, drug resistant variants [5], lack of resources and access to care in marginalized populations, and the inability to afford therapy in resource constrained nations [6]. Moreover, no infectious disease has ever been globally eradicated by therapy alone. Indeed Rinderpest and smallpox eradication were only possible through vaccination [7,8]. Even with a vaccine, eradication is not guaranteed: poliovirus continues to cause paralytic disease in several nations including Pakistan, Afghanistan, Nigeria, and Somalia [9]. Thus, with the new, highly effective DAA therapies, HCV will be controlled in wealthy countries in subjects that have access to care and the resources to afford therapy. However, due to the silent nature of acute hepatitis C and the slow, decades long progression of liver disease, millions of people in the world may be unaware they are chronically infected with HCV. Therefore, these individuals will not seek medical attention until they show signs of liver disease. These facts have led to recommendations from the Centers for Disease Control (CDC) in the USA to screen all baby boomers (*i.e.*, people born between 1945 and 1965) for HCV exposure [10]. Thus, there exists a significant global reservoir of undiagnosed chronic hepatitis C, and many individuals will unknowingly progress to liver disease. It is for these reasons that an understanding of how HCV causes inflammatory liver disease remains an unmet medical need.

## 4. Chronic Inflammation and the Road to HCV-Induced Liver Disease

Once HCV infects a hepatocyte, it is sensed by TLR3 [11] and RIG-I [12]. This leads to induction of the cellular antiviral (*i.e.*, production of IFN stimulated genes (ISGs) and pro-inflammatory (*i.e.*, production of inflammatory cytokines and chemokines) responses. Chemokines like CXCL8 [13] and CXCL10 [14] are produced by HCV-infected hepatocytes, resulting in recruitment of immune cells to the liver, which result in the killing of hepatocytes.

For example, activated CD8+ T cells, NK cells, and NK T cells kill virus-infected cells via several means such as Fas/TRAIL-mediated apoptosis, the release of granzymes and perforin, and secretion of type II IFN [15,16]. Kupffer cells, which are the liver-resident macrophages, take up apoptotic bodies released from dying hepatocytes, thereby accelerating hepatocyte death via Fas-mediated apoptosis [17]. Kupffer cells also secrete TGF-β, inducing differentiation of hepatic stellate cells into proliferative myofibroblasts, which then secrete type I collagen as part of the general wound healing response to liver injury [17]. This inflammatory response is also augmented by liver sinusoidal endothelial cells, which play important roles in antiviral and inflammatory responses to virus infection, by direct sensing of viral pathogen associated molecular patterns (PAMPS) [18]. In summary, the primary sensing of HCV RNA by hepatocytes engages anti-viral and pro-inflammatory responses that recruit multiple immune cell types to the site of infection to amplify the inflammatory response. Since the majority of acute HCV infections are not cleared, the inflammatory response is perpetuated.

## 5. Chronic Inflammation: The Case for HIV

Despite effective virus control by ART, many HIV+ patients live an average of 10–30 years less than the HIV-uninfected public [19]. A favored explanation is that this is due, in part, to dysregulated immune function that arises from chronic HIV infection. This chronic immune activation (CIA) is associated with various inflammatory diseases and both AIDS-defining and non-AIDS defining cancers [20]. One theory of how CIA manifests posits that the initial reduction in CD4 T cells in the gut releases PAMPs to trigger toll-like receptor (TLR)-mediated activation of immune cells [21].

By the end of this year, it is estimated that more than 50% of HIV-infected patients in the United States will be over the age of 50 [22]. In the aging HIV+ population, there are increases in co-morbidities such as osteoporosis, diabetes, and cardiovascular disease, which normally occur later in life due to natural aging [23,24]. Despite years of viral suppression by potent ART, a substantial proportion of HIV+ patients present with CIA, which is associated with disease progression and mortality [25]. Thus, while ART suppresses HIV replication, many patients’ CD4+ T cells fail to fully rebound to the level of non-infected patients [26], and this failure to is associated with a high rate of clinical events [27] and disease progression [28], and is thought to contribute to the shortened life expectancy in HIV infection [29] and accelerated aging. Immune-based factors, such as immunosenescence, characterized by continual immune cell death and turnover caused by uncontrolled, systemic, and low-grade inflammation, accelerates many of the aging phenotypes naturally observed in the HIV-negative elderly [30]: in HIV+ subjects with CD4 T cells that do not rebound during ART, their T cells are more likely to display immunosenescent phenotypes [31]; and these premature phenotypes are predictive of more rapid disease progression [32].

## 6. Metabolic Modulators That Impact Immune and Inflammatory Responses

An emerging body of literature has demonstrated the importance of cell metabolism in regulating the functionality and inflammatory status of the immune system [33,34,35]. In the following sections, we describe ways in which natural or synthetic drug modulation of key metabolic signaling enzymes influences immune cell function. We will focus on mTOR and AMPK, since they are key players in immune cell activation, inflammatory function, and fate decisions [34,36].

## 7. Immune Cell Activation

Immune cells exhibit marked changes in metabolism and metabolic signaling pathways when they transition from quiescence to activation. These metabolic changes also directly influence immune cell fate and functions. For example, resting/quiescent T cells produce energy in the form of ATP via the tricarboxylic acid (TCA) cycle and oxidative phosphorylation (OX PHOS) through the mitochondrial electron transport chain. This process requires the breakdown (*i.e.*, catabolism) of glucose, fatty acids, and amino acids, which are essential components for cell growth. In stark contrast, activated T cells engage the Warburg effect [37]; cells break down glucose by aerobic glycolysis to produce ATP. Even though glycolysis is an inefficient way to make ATP as compared to OX PHOS, the prevailing thought is that glycolysis is preferred for T cell activation because it is an anabolic (*i.e.*, cell building) form of metabolism which generates ATP while preserving amino acids, nucleotides, and fatty acids that are essential precursors for cell growth [38]. Exactly how these metabolic switches occur and how they affect immune cell activation and effector cell generation has become an area of active research [33,35,39,40].

## 8. Natural Products to Quell Chronic Inflammation in HCV and HIV Infection

**Figure 1:**
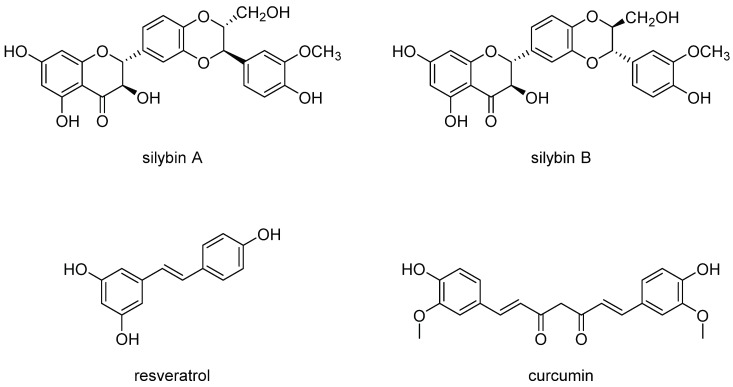
Structures of silybin A and silybin B, resveratrol, and curcumin. Note that silybin A and silybin B comprise the two major flavonolignans in the extract known as silymarin. Silibinin is an equimolar mix of silybin A and silybin B. Not shown are the other 5 flavonolignans that comprise the extract silymarin [44]. The structures share the features of containing multiple phenol groups. Hence, these compounds are all polyphenol-containing natural products.

There is a huge body of literature on many natural products and how they impact cellular inflammation and inflammatory signaling pathways. The reader is referred to recent reviews on the subject [41,42,43]. The following discussion focuses on three structural distinct polyphenolic compounds: curcumin, resveratrol, and silymarin (Figure 1). To assist with making certain points, we will also describe the actions of a few other natural products.

Curcumin is a diarlyheptanoid, distantly related to flavonoids because it possesses two phenol moieties and is therefore a polyphenolic natural product. It is derived from the rhizomes of *Curcuma longa*, a plant in the ginger family. It has diverse anti-inflammatory actions including direct suppression of pro-inflammatory cytokines and chemokines such as TNF-α, IL-1β, and CXCL8 [42]. Curcumin also appears to activate antioxidant responses by activation of the transcription factor Nuclear factor (erythroid-derived 2)-like 2 (Nrf2), which induces the expression of antioxidant genes including heme oxygenase 1, glutathione S-transferases (GST), and NAD(P)H quinone oxidoreductase 1. The pathways that account for these effects are diverse and include modulation of transcription factors including NF-κB, β-catenin, and Signal Transducer and Activator of Transcription 3 (Stat3) [42].

Resveratrol is a polyphenolic natural product (a stilbene) found in several plant species including red grapes. Resveratrol is thought to interact with several different putative cellular protein targets including Sirt1, cAMP phosphodiesterases, F1-ATPase [43]. Like curcumin, resveratrol shows strong anti-inflammatory activity, including suppression of COX2, NF-κB, and FOXO action [43].

Silymarin is an extract derived from the seeds of the milk thistle plant *Silybum marianum*, which is part of the Daisy family (Asteraceae). Silymarin is one the most popular natural products consumed by HCV-infected subjects [45], and is consumed by HIV-infected subjects [46]. Silymarin blocks HCV infection *in vitro* [47,48,49,50,51], and an intravenous formulation of silibinin (a major component of silymarin, known as silibinin succinate), inhibits HCV replication in humans [52,53,54,55]. Furthermore, silibinin succinate also inhibits HIV-1 infection *in vitro* [56].

Silymarin shows broad-spectrum anti-inflammatory activity, which, in addition to its antiviral activity, may have direct relevance for both HCV- and HIV-induced inflammation. In fact, there is evidence that the anti-inflammatory activity of silymarin occurs at various levels and targets multiple pathways. First, silymarin inhibits pro-inflammatory signaling pathways including NF-κB [47,48,57,58]. Second, silymarin inhibits the expression of multiple pro-inflammatory cytokines and chemokines (e.g., CXCL1, CXCL2, CXCL8, CXCL10, IL-1, TNF-α [57]), all of which are dysregulated in CIA and during HIV-1 disease progression. Third, silymarin suppresses T-cell activation and proliferation [47,48,58], which is elevated in HIV-associated CIA.

Exemplified by the three compounds described above, a common theme among hundreds of polyphenolic natural products is that they almost invariably suppress inflammation (albeit it to differing degrees depending on the structure of the compound, the experimental system, dose, and duration of exposure). For example, at least a dozen natural products have been shown to effectively inhibit inflammatory bowel disease in mouse models through suppression of NF-κB and NF-κB regulated cytokines and chemokines (reviewed in [41]). This raises the question of how can so many structurally diverse compounds cause the same effects in cells? As far as defining the mechanistic underpinnings for the anti-inflammatory actions of natural products, there are plenty of examples of many natural products that inhibit a particular pathway, kinase, transcription factor, or modulate cellular gene and protein expression. There is also the frequent misconception in the literature that inhibition of an inflammatory pathway indicates that pathway is the target (*i.e.*, the natural product binds to the protein). While many natural products do have bona fide protein targets to which they bind, the interaction may occur far upstream of the measured biological effect. Thus, there is great imprecision and a lack of a unified view of explaining how natural products suppress inflammation. As we shall see in the next section, the unifying effects that explain the anti-inflammatory effects of many natural products may lie at the level of altering cellular metabolism and cellular signaling pathways that receive metabolic inputs and cues.

## 9. AMPK, and mTOR: Cellular Signaling Pathways That Link Cellular Metabolism to Function

**Figure 2:**
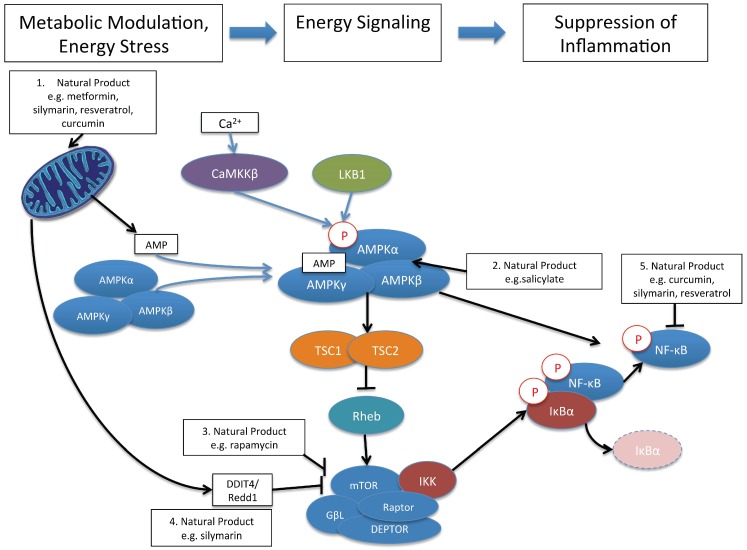
Metabolic Rules of Engagement of Natural Products with Cells. Some of the earliest cellular responses to natural product exposure include the alteration of mitochondrial function which increases the AMP:ATP ratio, leading to activation of AMPK by phosphorylation (1); Natural products like salicylate can also directly bind to and activate AMPK (2); AMPK activation suppresses Rheb, an upstream activator of mTOR signaling. Thus, AMPK activation by natural products can lead to mTOR inhibition. Natural products like rapamycin can also directly bind to and inhibit mTOR signaling (3); The alteration of mitochondrial energetics may be involved in natural product induction of DDIT4, a novel upstream inhibitor of mTOR (4); Finally, activation of AMPK and inhibition of mTOR by natural products may be directly involved in the suppression of pro-inflammatory signaling such as that mediated by NF-κB (5). For mTOR, there is evidence that IKK interacts with mTORC1 to mediate the connection of mTORC1 to NF-κB, while for AMPK, the route to NF-κB is not presently clear. Citations of studies supporting these interactions are provided in text. Note that the tri-molecular complexes of IKK (IKKα, IKKβ, and IKKγ), and the bimolecular complex of NF-κB are simplified as single proteins. Moreover, only the mTORC1 and canonical NF-κB pathways are shown.

Mammalian cells express sentinel kinases and transcription factors that sense nutritional status in the microenvironment. For example, AMPK is a master regulator of cellular metabolism. AMPK is a tri-molecular complex of a catalytic α subunit (with serine/threonine kinase function) and regulatory β and γ subunits [59] (Figure 2). AMPK is activated by phosphorylation on Thr-172 by two major routes. One is via an upstream kinase known as Serine/threonine kinase 11 (STK11; a.k.a. liver kinase B1 (LKB1)), while the other upstream kinase is the calmodulin-dependent kinase kinases, CaMKKβ. LKB1 activates AMPK by phosphorylation in response to energy stress (*i.e.*, decreased cellular ATP/ADP ratios), while CaMKKβ activates AMPK in response to treatments that increase intracellular Ca^2+^ (Figure 2). Thus, AMPK is regulated by adenine nucleotides and calcium ions. The downstream effects of AMPK activation are numerous and serve to realign cellular energetics; more specifically, to restore the reductions in cellular ATP. Thus, catabolic responses (*i.e.*, energy sparing) are activated while anabolic (*i.e.*, energy consuming/cell building) processes are curtailed. Downstream effects occur through multiple transcription factors and changes in cellular metabolism including increasing glucose uptake, glycolysis, fatty acid uptake, fat oxidation, and autophagy, and to decrease anabolic processes such as protein, cholesterol, and fatty acid synthesis [59].

The mTOR pathway [60,61] is one downstream pathway that is inhibited by activated AMPK [62]. mTOR is a major hub for several metabolic inputs and cues, and consists of two complexes, mTOR containing complex 1 and 2 (mTORC1 and mTORC2). By sensing energy status, mTOR affords switching between anabolic (in a nutrient-rich environment) to catabolic (during nutrient depletion/stress) processes. Thus, when nutrients are abundant, mTOR kinase activity leads to the phosphorylation of several downstream targets that promote cell growth and survival, including mRNA and protein synthesis. mTOR complex 1 (mTORC1) consists of mTOR, GβL, DEPTOR and Raptor [63,64] and is regulated by a small GTPase, Rheb, which when bound by GTP, potently activates mTORC1 kinase activity. mTOR complex 2 (mTORC2) is composed of mTOR, Rictor, GβL, Sin1, PRR5/Protor-1, and DEPTOR [65,66]. mTORC2 promotes cellular survival by activating Akt and other pathways [67]. Thus, for the purpose of this review, natural products can be viewed as metabolic modulators that activate AMPK and suppress mTOR, which ultimately converge of suppression of pro-inflammatory signaling, exemplified by blockade of NF-κB (Figure 2).

Nuclear factor kappa B (NF-κB) is the classical pro-inflammatory transcription factor that controls the expression of hundreds of pro-inflammatory cytokines and chemokines as well as other inflammatory and immune molecules such major histocompatibility complex proteins, transcription factors, and inhibitors of apoptosis [68]. NF-κB is not a single protein but rather a complex of homo and heterodimeric proteins [69]. Inactive NF-κB maintains a cytoplasmic localization by associating with inhibitory proteins of the IκB family [70]. Stimulation of cells via cytokines, PAMPs, or immune cell receptor activation leads to NF-κB activation by phosphorylation of IκB proteins, which leads to their degradation (Figure 2). This allows NF-κB to enter the nucleus and exert its transcriptional effects. IκB phosphorylation is controlled by the multiprotein IκB-kinase (IKK) complex that contains 2 catalytic subunits, IKKα (IKK1) and IKKβ (IKK2), as well as the regulatory subunit IKKγ (a.k.a NEMO for NF-κB essential modifier). There are two pathways that lead to NF-κB activation: canonical and alternate. Activation of NF-κB via the canonical pathway involves phosphorylation of IκB-α by the IKKα/IKKβ/IKKγ complex, followed by degradation of IκB-α, permitting NF-κB (as p65/cRel and p50 dimers) to translocate to the nucleus (Figure 2). The alternate pathway involves another upstream molecule, NF-κB inducing kinase (NIK), which activates IKKα homodimers, which phosphorylate NF-κB (as a RelB and p100 dimer), leading to nuclear translocation [71,72].

## 10. Control of Immune and Inflammatory Responses by Natural Product Modulation of Metabolic Signaling by AMPK and mTOR

Galegine (related to guanidine), a natural product from the plant *Galega officinalis* is a potent AMPK activator used in the treatment of diabetes in the early part of the 20th century. Metformin (dimethylbiguanidine), also a potent AMPK activator, was subsequently developed as an anti-diabetic drug and is still used widely today. AMPK activation and the subsequent increased uptake of glucose, and reduction of fatty acid and triglyceride synthesis might be involved in how metformin improves insulin sensitivity. Interestingly, metformin activates AMPK by increasing the cellular ADP:ATP ratio, and this appears to arise through suppression of mitochondrial function [73].

In terms of AMPK’s role in modulating immunity, a recent study found that metformin maintains anti-tumor CD8 T cells [74]. Specifically, metformin increased the number of CD8+ tumor infiltrating lymphocytes (TIL), which were found to be resistant to apoptosis and exhaustion, *i.e.*, the cells were mainly PD-1 negative and Tim 3 positive, which are markers of tumor-rejecting effector memory cells. Thus, metformin supported the number and functionality of T cells in the tumor microenvironment, where T cells often become exhausted and therefore cannot act against tumor cells.

Rapamycin was discovered on the Pacific Island of Rapa Nui in 1975 [75]. Although originally discovered as a novel antifungal agent, experiments on mammalian cells revealed rapamycin interacts with protein FK-binding protein 12 (FKBP12) [61] to inhibit mTOR [76]. While primarily used as an immunosuppressant, recent data highlight the compound’s ability to alter inflammation and immune responses in novel and exciting ways. For example, administration of rapamycin during immunization of mice with influenza virus completely changed the immune response in favorable ways [35]. Specifically, rapamycin altered the B cell response, resulting in a novel repertoire of antibodies that provided cross-strain protection against lethal influenza infection. A different mTOR inhibitor, RAD001 (Everolimus, PubChem Compound Identification number CID 6442177), was shown to increase the response to the influenza vaccine in elderly individuals. In this study, RAD001 treatment was associated with a reduction in the number of CD4 and CD8 T lymphocytes expressing the programmed death-1 (PD-1) receptor, which inhibits T cell signaling and is more highly expressed with age [77]. Furthermore, mTOR inhibition by rapamycin stimulates the production of memory CD8+ T cells in both murine and non-human primate infection and vaccination experiments [33]. These data suggest that modulation of cellular metabolism via mTOR is able to enhance immune responses to vaccine- or infection-induced challenges.

In addition to suppressing inflammation in many *in vitro* cell culture and *in vivo* animal models, natural products like curcumin, resveratrol and silymarin also share the function of regulating AMPK and mTOR. For example, resveratrol treatment of multiple cell types activates AMPK [43], although this effect, associated with decreased ATP levels, tends to be observed only with high concentrations of resveratrol (>50 μM) [78,79]. These data suggest that at high concentrations, resveratrol inhibits mitochondrial function, reducing cellular ATP levels, leading to AMPK activation (Figure 2). Curcumin also appears to regulate mitochondrial function when the compound induces apoptosis of tumor cells [80]. Of note, resveratrol also regulates cyclic AMP levels, which can regulate AMPK activation by distinct mechanisms. The reader is referred to a recent review for further details [43].

Silibinin, which is a mixture of silybin A and silybin B (Figure 1), and is the major component of silymarin, suppresses cellular metabolism by reducing glycolysis and mitochondrial function [81,82,83,84]. We recently discovered that silymarin treatment also alters cellular metabolism by reducing glycolysis and mitochondrial respiration [81], and metabolomics analyses demonstrate that silymarin suppresses glycolytic, tricarboxylic acid (TCA) cycle, and amino acid metabolism [57]. Moreover, modulation of cellular metabolism by silymarin involves regulation of multiple metabolic pathways including activation of adenosine monophosphate kinase (AMPK) and suppression of mammalian target of rapamycin (mTOR) ([57]; Figure 2). The activation of AMPK by silymarin may be indirect via increased AMP:ATP ratios that arise through suppression of glycolysis and mitochondrial function. However, since relatively high doses of silymarin were required to show AMPK activation, it is possible that other mechanisms are involved in AMPK activation by silymarin. In terms of silymarin suppression of mTOR, we showed that silymarin induces DDIT4/Redd1, a novel inhibitor of mTOR that is normally induced during hypoxia [85]. Using mouse knock out cells, suppression of mTOR by silymarin was found to be partially dependent on DDIT4 and AMPK [57] (Figure 2). Thus, in contrast to the direct suppression of mTORC1 by rapamycin binding to mTOR, silymarin may inhibit mTOR via at least 2 pathways: suppression of mitochondrial function to activate AMPK to suppress mTOR and direct induction of DDIT4 to suppress mTOR.

Energy sensing pathways like AMPK and mTOR communicate to inflammatory pathways. For example, activation of AMPK by metformin and the synthetic compound 5-Aminoimidazole-4-carboxamide riboside (AICAR) inhibits TNF-induced activation of NF-κB dependent pro-inflammatory cytokine production [86] (Figure 2). As described above, silymarin activates AMPK and also inhibits NF-κB-dependent transcription. In murine cells expressing wild type AMPK, silymarin inhibited TNF-α induced NF-κB transcription, as expected. However, in cells with a double knockout of AMPKα1 and α2 subunits, silymarin inhibition of NF-κB transcription was attenuated [57]. Thus, silymarin suppression of inflammatory signaling through the NF-κB pathway was dependent on AMPK. Similar correlations (*i.e.*, without causal connections) between pathways have been shown for curcumin and resveratrol, where natural product activation of AMPK was associated with inhibition of NF-κB phosphorylation [87,88]. Additional research is required to define the steps from AMPK activation to suppression of NF-κB transcriptional activity.

mTOR and NF-κB pathways are also similarly connected. For example, activation of mTOR signaling by AKT leads to activation of NF-κB, and this appears to involve an association of IKK with Raptor within the mTORC1 complex [89]. Similarly, suppression of mTOR by curcumin, silymarin, and resveratrol correlates with suppression of NF-κB activity [57,90,91]. Thus, multiple natural products alter cellular metabolism and metabolic signaling pathways like AMPK and mTOR, and we propose that the modulation of these and likely other pathways culminate in the anti-inflammatory cellular phenotype observed with silymarin treatment.

*Where do we go from here?* There remain several pressing questions that are common to most if not all studies on natural products that have anti-inflammatory action.

*What about the metabolism of natural products?* Most herbal natural products are consumed orally as extracts, which, after metabolism and uptake in the intestine, get transported via the portal vein into the liver. Upon uptake by hepatocytes (the predominant cell type in the liver), herbal extracts are first modified by Phase I reactions that involve oxidation, reduction, and hydrolysis by cytochrome P450 enzymes. Phase II metabolic enzymes that perform conjugation reactions further alter natural products: transfer of methyl, sulfur, acetyl, or glucuronide moieties. Finally, in Phase III metabolism, compounds are further modified and excreted from the cell for eventual elimination from the body via the kidneys or gastrointestinal system. Thus, the adsorption, distribution, metabolism, and excretion (ADME) define the extent and duration of exposure of cells to natural products. While many herbal natural products are metabolized and excreted from the body within 2–8 h [42,92], there are many examples of beneficial drugs that have similarly short half-lives *in vivo* including ibuprofen, benadryl, and acetaminophen.

*Considerations:* Natural products like flavonoids possess biological activity that arises due to interactions with other, non-protein cellular components and/or inherent chemical properties. For example, natural products like curcumin and resveratrol were recently shown known to cause similar effects on membrane protein functions based on their interaction with the bilayer/solution interface of cell membranes [93]. Some natural products can also exhibit the phenomenon known as PAINS (pan assay interference compounds [94]), which typically involve reactive compounds that produce false readouts in *in vitro* assays, the majority of which are biochemical and non-cell based. For example, resveratrol was originally reported as an activator of Sirtuin 1, a protein involved in prolonging lifespan [95]. However, subsequent studies found that the activation in the screening assay was due to interaction between resveratrol and the fluorescent reporter substrate [96,97,98]. On the other hand, a recent study shows that the Rodgersinine Family of 1,4-benzodioxane neolignan polyphenolic natural products display anti-HCV activity [99]. This result is important since the rodgersinines lack the chromanone (*i.e.*, flavonoid core), indicating that biological activity is not solely dependent on flavone moieties, which have been suggested to be responsible for PAINS activity. Thus, while PAINS activity can occur in non-cellular systems, many natural products reproducibly evoke metabolic and anti-inflammatory effects in biological systems. Regarding chemical properties, both synthetic drugs and natural products have been shown to produce aggregates or colloids, which often form at or below the concentration of compound which shows biological activity [100]. Colloid formation promotes the adsorption of soluble proteins to the surface of the colloid, which can lead to denaturation and nonspecific effects on cellular proteins. For *in vitro* enzymatic reactions with purified proteins, aggregate activity can be neutralized by addition of detergent [101]. Furthermore, non-toxic concentrations of detergent can be added to cells in culture, which has shown to effectively disperse colloids into monomers in solution [102]. Thus, considerations of PAINS activity, aggregate formation, and assay context (non-cell based versus bioassay) must be incorporated into any natural product research program that seeks to use natural products as tools to define the biology of inflammation. While many natural product studies have been focused on the protein interaction/signaling hypothesis, the ideal approach should also consider the role of natural product-membrane and natural product chemical properties in mediating these diverse biological effects.

Collectively, the data described above suggest that modulating cellular metabolism via key checkpoints such as AMPK and mTOR provide novel opportunities for altering immune and inflammatory responses. While both AMPK and mTOR are highlighted in this review, many compounds derived from natural products perturb many pathways to achieve similar anti-inflammatory phenotypes. Therefore, as research continues to delve into controlling deregulated immune states, whether they be in the context of an ongoing, uncontrolled, chronic infection or when an infection has been cleared or suppressed but the immune system has not recovered, it is clear that one possible avenue to reigning in and quenching this deregulation is through modulating the metabolism of the target cells. When the diverse chemical properties of natural products are properly considered, these compounds represent a diverse class of novel and powerful tools for defining how to suppress inflammation in hepatocytes or non-parenchymal cells (in the context of HCV-induced liver disease) and in immune cells (that contribute to chronic inflammation during HIV infection).
